# The Elimination of the Middle Class

**Published:** 1914-02-14

**Authors:** 


*To the Editor of* The Hospital.

### The Elimination of the Middle Class.

*To the Editor of* The Hospital.

Sir,—Your correspondent on the subject of the elimination of the middle class voiced, I think, the opinion of many. As the mother of nine, and belonging as I do to that much suffering community, I venture to have views upon the subject of education. This is an appalling problem to the middle class in this country, more especially to those who have done their duty to the State—that is, as the State understands by duty, a large family.

Monetary losses occurring somewhat early in my married life led my husband to accept a post in a large New Zealand town. Here we went with five children, all to be educated. At once looking around for suitable schools for slender means, I found to my surprise that everybody sent their children to the State-supported schools, the well-to-do and all. Thus my children received a good education and no stigma attached.

Later on the family increased, but by this time our home was in Scotland. As everyone knows, education is better understood in Scotland than anywhere else. A large endowed school, of which there are so many there, was where the children were sent. Residence close to the school gave great facilities as to fees, and here the paying and unpaying sat side by side and received an education of a first-class quality. Boys and girls are taught together, and the washerwoman's son sits next to the laird's daughter with no evil result.

Thus my nine passed into the world well equipped at little cost with a good education, though little else of worldly advantages, and fill professional careers to-day with credit. I do not think that this could have been done in England. Is it not right that the State should give something in the way of help in the matter of education to those who are willing to give the best that a nation can receive at its hands—namely, the gift of citizens? At the present rate of taxation this is becoming more and more of an impossibility. If the middle class be the backbone of the country, surely it is well worth preserving.—I am, Sir, E. M. H. B.

